# Risk factors for ductal and lobular breast cancer: results from the nurses' health study

**DOI:** 10.1186/bcr2790

**Published:** 2010-12-08

**Authors:** Joanne Kotsopoulos, Wendy Y Chen, Margaret A Gates, Shelley S Tworoger, Susan E Hankinson, Bernard A Rosner

**Affiliations:** 1 Channing Laboratory, Department of Medicine, Brigham and Women's Hospital and Harvard Medical School, 181 Longwood Avenue, 3rd Floor, Boston, MA 02115, USA; 2 Department of Medical Oncology, Dana-Farber Cancer Institute, 44 Binney Street, Boston, MA 02115, USA; 3 Department of Epidemiology, Harvard School of Public Health, 677 Huntington Avenue, Boston, MA 02115, USA; 4 Department of Biostatistics, Harvard School of Public Health, 655 Huntington Avenue, SPH2, 4th Floor, Boston, MA 02115, USA

## Abstract

**Introduction:**

Ductal and lobular carcinomas are the two most common types of invasive breast cancer. Whether well-established risk factors are differentially associated with risk on the basis of histologic subtype is not clear. We prospectively investigated the association between a number of hormonal and nonhormonal exposures and risk defined by histologic subtype among 4,655 ductal and 659 lobular cases of postmenopausal breast cancer from the Nurses' Health Study.

**Methods:**

Multivariate Cox proportional hazards regression stratified by histologic subtype and time period was used to examine the association between risk factors and the incidence of ductal and lobular subtypes. For each exposure, we calculated the *P *value for heterogeneity using a likelihood ratio test comparing models with separate estimates for the two subtypes versus a single estimate across subtypes.

**Results:**

The associations with age at menarche (*P*-heterogeneity (het) = 0.03), age at first birth (*P*-het < 0.001) and postmenopausal hormone use (*P*-het < 0.001) were more strongly associated with lobular cancers. The associations with age, nulliparity, parity, age at menopause, type of menopause, alcohol intake, adult body mass index (BMI), BMI at age 18, family history of breast cancer and personal history of benign breast disease did not vary by subtype (*P*-het ≥ 0.08). Results were similar when we restricted the analyses to estrogen receptor-positive and progesterone receptor-positive tumors.

**Conclusions:**

These data indicate that breast cancer is a heterogeneous disease, and the differential association with a number of risk factors is suggestive of etiologically distinct tumors. Epidemiological analyses should continue to take into account a modifying role of histology.

## Introduction

Epidemiologic studies have shown that reproductive, lifestyle and anthropometric exposures are predictive of subsequent breast cancer risk [1,2]; however, whether these factors are differentially associated with risk on the basis of histologic subtype is not clear. Among invasive breast cancer cases, ~75% are ductal carcinomas, 15% are of the lobular type and the remainder are a mix of other, less common histologies [3]. Differences in clinical, molecular and pathologic features of ductal and lobular tumors [4] suggest a distinct etiology and may have implications with respect to their prevention, diagnosis and treatment. Further, lobular cancers are more likely than ductal cancers to be estrogen receptor (ER)-positive and progesterone receptor (PR)-positive [5,6]. This might explain why several studies have demonstrated differences in the association between postmenopausal hormone use (PMH) and risk of breast cancer by histology, with a stronger effect seen for lobular carcinomas [7].

Besides the studies of PMH and breast cancer risk by histology, few large studies have evaluated whether the association of other well-established breast cancer risk factors varies by histology [8-10]. Moreover, limitations of prior studies include the lack of formal statistical testing across disease subtypes, small sample size, no control for ER or PR status or the use of retrospective data. Therefore, we prospectively examined the relationship between known breast cancer risk factors and their association with the two most common types of invasive breast cancers, ductal and lobular, diagnosed among postmenopausal women from the Nurses' Health Study (NHS) cohort.

## Materials and methods

### Study population

The NHS was initiated in 1976, when 121,700 female registered nurses between the ages of 30 and 55 and residing in 11 U.S. states completed a self-administered, mailed questionnaire about their medical histories and health-related exposures [11-13]. Every 2 years, follow-up questionnaires have been mailed to update information on risk factors and disease development. A food frequency questionnaire was added in 1980, and subsequent dietary questionnaires were distributed in 1984, 1986 and every 4 years thereafter. We defined baseline as 1980, the first time we assessed alcohol intake (*n *= 92,468). Follow-up has been high in this cohort (95.2% of the total possible person-years through June 2006). The study protocol was approved by the Institutional Review Board at Brigham and Women's Hospital.

### Exposure data

We obtained information on the exposures of interest from one or more of the biennial questionnaires completed by participants. On the baseline questionnaire, we collected information on date of birth, age at menarche, weight at age 18 and current height. The baseline and all subsequent questionnaires requested information on status and duration of PMH use, menopausal status, type of menopause, history of benign breast disease (BBD) and current weight as well as, beginning in 1978, the type of PMH.

For the other exposures of interest, questions were included on multiple but not all questionnaires, and we updated the value for each covariate when new information was available or otherwise carried forward the value from the previous follow-up cycle. For the exposures that were collected at a single time point (for example, age at menarche), we carried forward the value throughout the remainder of follow-up. Age at menopause was requested on all questionnaires through 2000 and menopausal status was included on all questionnaires through 2002, at which time the youngest participants were 55 years of age. We assessed age at first birth (AFB) and parity on every NHS questionnaire until 1984 and again in 1996 to update the data on each woman's lifetime pregnancy history. Data on family history of breast cancer in a first-degree relative were collected every 4 to 8 years beginning in 1976. Daily alcohol consumption was first queried in 1980, then in 1984 and 1986 and every 4 years thereafter.

### Identification of breast cancer cases

The primary end point was the diagnosis of invasive ductal or invasive lobular breast cancer. Carcinomas *in situ *were censored. On each questionnaire, we asked whether breast cancer had been diagnosed and, if so, the date of diagnosis. We asked all women who reported breast cancer (or next of kin for those who had died) for written consent to review the pertinent medical records for confirmation. We also searched the National Death Index for breast cancer deaths among women who did not respond to the questionnaires, which accounted for less than 1% of confirmed breast cancer cases. Pathology reports or cancer registry data were obtained for 95% of the cases. Histologic type and ER or PR status were based upon extraction from the medical records. Invasive tumors of mixed ductal and lobular type or of other or unknown histology were censored at the date of diagnosis.

### Exclusions

We excluded the following women at baseline from the analysis: those reporting a previous diagnosis of ductal carcinoma *in situ *(DCIS; *n *= 26) or any invasive cancer except nonmelanoma skin cancer (*n *= 4,933), those reporting no year of birth (*n *= 161) and those who died before 1980 (*n *= 461). Women were censored at the time of development of DCIS or any type of invasive cancer except nonmelanoma skin cancer. Only postmenopausal women were included in the analysis, because the analysis included many hormonal factors that may operate differently in pre- and postmenopausal women, and we had limited numbers of woman with premenopausal lobular breast cancers. Women who were premenopausal at baseline began contributing person-time to the analysis at the time that they became postmenopausal. Menopause was defined as natural, radiation-induced (very few women had radiation-induced menopause: ~0.14%) or surgical (hysterectomy with or without unilateral or bilateral oophorectomy).

### Statistical analysis

Participants accrued person-time from the return date of the baseline questionnaire until the date of breast cancer diagnosis, diagnosis of any other cancer (excluding nonmelanoma skin cancer), death or the end of follow-up (2006). Participants contributed person-time only for follow-up periods for which they were postmenopausal. For the reproductive and hormonal exposures, we modeled age, age at menarche and age at menopause as continuous variables to minimize the number of parameters in the model. We used categorical variables to model type of menopause (natural or radiation-induced, surgical with two ovaries removed, surgical with one or zero ovaries removed and unknown), nulliparous or parous, AFB (AFB <25 years, AFB 25-29 years, AFB 30-34 years, AFB ≥35 years, parity (1, 2, 3, or ≥4) and PMH use (never, past user, current estrogen (E) <5 years, current estrogen and progesterone (E + P) <5years, current other ≤10 years, current E 5-10 years or current E+P 5-10 years). For analyses of duration of PMH use by type of PMH, we included only PMH use durations of ≤10 years because PMH use longer than 10 years was for the most part limited to E-alone formulations. PMH use for women with use for >10 years was censored. For the nonreproductive exposures, we modeled recent body mass index (BMI) (<21, 21 to <23, 23 to <25, 25 to <30 or ≥30 kg/m^2^), BMI at age 18 (<19, 19-21, 21 to <23 or ≥23 kg/m^2^) and alcohol consumption (0, <5, 5 to <15 or ≥15 g/day) as categorical variables. Continuous variables were used to assess *P *trend. Family history of breast cancer in a first-degree relative and personal history of BBD were modeled as binary variables (yes or no). We carried forward the last self-reported exposure data for one cycle if the data were missing. Otherwise, women with missing information were included in the analysis using indicators for the missing variables. Findings were similar in analyses including only nonmissing data (complete case analysis).

We used Cox proportional hazards regression stratified by time period to model the incidence rate ratio (RR) and 95% confidence interval (CI) of invasive cancer overall for each exposure. We then restricted the analysis to cases with ductal or lobular histology and used Cox proportional hazards regression stratified by type of outcome and time period to allow for different associations by histologic subtype [14]. We used data augmentation, such that each participant had a separate observation for each subtype. We coded the event variable as 1 (failed) if the participant was diagnosed with the histologic subtype corresponding to that data row, and as 0 otherwise; cases were censored for the other subtypes at the time of diagnosis.

We compared a model that assumed different associations for all exposures by histologic subtype (full model) to a model with a single estimate across histologic subtypes for one exposure at a time (reduced model). We calculated the *P *value for heterogeneity (*P*-het) using a likelihood ratio test, with the degrees of freedom equal to the difference between the number of parameters in the full and reduced models. Using a stepwise-down approach, we set exposures with a nonsignificant *P*-het (*P *> 0.05) to have a single parameter estimate across subtypes so that the final model estimated two separate associations for exposures that differed significantly by subtype and a single parameter estimate for all other exposures. In the current analysis, we show the results from the model that allowed all the associations to vary (full model). In secondary analyses, we also examined these associations limited to ER-positive and PR-positive tumors.

All analyses were conducted using SAS version 9.1 software (SAS Institute, Cary, NC, USA). All *P *values were based on two-sided tests and were considered statistically significant if *P *≤ 0.05.

## Results

At baseline, our analysis included 37,127 postmenopausal women and then expanded to 107,759 women by the end of follow-up. During 1,774,075 person-years of follow-up, we observed 6,226 incident cases of invasive breast cancer. Of these, 4,655 were ductal and 659 were lobular carcinomas (see Table 1 for characteristics by case type). The remaining 1,002 were excluded because of no pathology report (8%), no primary structure evident (1%), mixed ductal and lobular histologies (4%) or unknown or other histology (1%).

**Table 1:** Baseline characteristics of invasive postmenopausal breast cancer cases and noncases in the Nurses' Health Study in 1980

	Noncases (*n *= 101,533)	All invasive cases (*n *= 6,226)	Ductal carcinoma (*n *= 4,655)	Lobular carcinoma (*n *= 659)
Reproductive or hormonal characteristics				
Mean age (yr)	53.7	54.5	54.5	54.4
Mean age at menarche (yr)	12.7	12.6	12.6	12.5
Mean parity among parous women	3.3	3.2	3.2	3.3
Mean age at menopause (yr)^a^	48.7	49.5	49.4	49.7
Ever used postmenopausal hormones (%)	47	48	50	51
Natural menopause (%)	64	67	67	66
Nonreproductive characteristics				
Mean body mass index (kg/m^2^)	24.9	25.2	25.2	24.5
Mean body mass index at age 18 (kg/m^2^)	21.4	21.0	21.0	21.2
Median alcohol intake (g/day)	1.8	1.8	1.8	1.8
Family history of breast cancer in first degree relative (%)^b^	8	13	14	11
History of benign breast disease (%)	22	27	27	30

^a^Among those who experienced natural menopause. ^b^Family history in 1982.

### Reproductive and hormonal exposures

There was no heterogeneity in the risk estimates for age, nulliparity, parity, age at menopause or type of menopause by histology (*P*-het = 0.18, 0.87, 0.35, 0.45 and 0.08, respectively) (Table 2). Although an inverse association with surgical menopause was observed for ductal but not lobular cancers, the *P*-heterogeneity value was not significant.

**Table 2:** Association between various reproductive and nonreproductive exposures and risk of postmenopausal breast cancer by histologic subtype among 107,759 women in the Nurses' Health Study^a^

	Ductal (*n *= 4,655)		Lobular (*n *= 659)		
Reproductive or hormonal exposures	Cases (n)	RR (95% CI)	Cases (n)	RR (95% CI)	***P*-heterogeneity**^**b**^
Age (per year)^c^	4,655	1.03 (1.03-1.04)	659	1.04 (1.03-1.06)	0.18
Age at menarche (per year)^c^	4,655	0.98 (0.96-1.00)	659	0.92 (0.87-0.97)	0.03
Parous	4,220	1.00 (ref)	610	1.00 (ref)	
Nulliparous	437	1.26 (1.12-1.41)	49	1.22 (0.87-1.71)	0.87
Age at first birth (AFB)					
AFB < 25	2,354	1.00 (ref)	268	1.00 (ref)	
AFB 25-29	1,741	1.11 (2.04-1.19)	282	1.63 (1.37-1.96)	
AFB 30-34	425	1.21 (1.09-1.35)	86	2.31 (1.79-2.99)	
AFB ≥35	137	1.31 (1.10-1.57)	23	2.10 (1.35-3.26)	<0.001
AFB, continuous, RR (*P *trend)^c^		1.12 (<0.001)		1.48 (<0.001)	<0.001
Parity					
Parity = 1	813	1.00 (ref)	100	1.00 (ref)	
Parity = 2	1,171	0.99 (0.91-1.07)	180	0.94 (0.76-1.16)	
Parity = 3	1,269	1.04 (0.96-1.13)	163	0.88 (0.71-1.09)	
Parity ≥ 4	1,404	0.91 (0.82-1.00)	216	0.96 (0.75-1.24)	0.35
Parity, continuous, RR (*P *trend)^d^		0.97 (0.01)		1.05 (0.11)	0.02
Postmenopausal hormone use					
Never used	1,616	1.00 (ref)	181	1.00 (ref)	
Past use	1,165	1.05 (0.97-1.14)	150	1.11 (0.88-1.39)	
Current use, estrogen alone < 5 years	143	1.07 (0.90-1.28)	24	1.56 (1.01-2.42)	
Current use, estrogen plus progesterone < 5 years	283	1.56 (1.36-1.78)	42	2.16 (1.52-3.06)	
Current use, other ≤10 years	267	1.31 (1.15-1.49)	46	1.95 (1.40-2.71)	
Current use, estrogen alone 5-10 years	518	1.30 (1.17-1.45)	93	1.76 (1.34-2.34)	
Current use, estrogen + progesterone 5-10 years	463	1.75 (1.57-1.95)	99	3.12 (2.41-4.05)	<0.001
Estrogen alone, continuous, RR (*P *trend)^e^		1.00 (0.008)		1.00 (0.24)	1.00
Estrogen + progesterone, continuous, RR (*P *trend)^e^		1.02 (<0.001)		1.03 (<0.001)	0.43
Other, continuous, RR (*P *trend)^e^		1.00 (0.45)		1.01 (0.30)	0.52
Age at menopause (per year)^c^	4,655	1.01 (1.00-1.01)	659	1.01 (1.00-1.02)	0.45
Menopausal type					
Natural or radiation-induced	2,958	1.00 (ref)	391	1.00 (ref)	
Surgical with two ovaries removed	667	0.80 (0.73-0.89)	101	1.00 (0.78-1.29)	
Surgical with one or no ovaries removed	559	0.87 (0.79-0.96)	100	1.21 (0.95-1.53)	0.08
Nonreproductive exposures					
Body mass index (BMI)					
<21	473	1.00 (ref)	70	1.00 (ref)	
21 to less than 23	719	1.15 (1.02-1.29)	104	1.11 (0.82-1.50)	
23 to less than 25	858	1.21 (1.08-1.35)	122	1.14 (0.85-1.54)	
25 to less than 30	1,590	1.41 (1.27-1.57)	225	1.33 (1.01-1.75)	
≥30	926	1.60 (1.42-1.80)	124	1.47 (1.08-2.00)	0.20
BMI, continuous, RR (*P *trend)^f^		1.14 (<0.001)		1.12 (0.009)	0.75
BMI at age 18					
<19	839	1.07 (0.98-1.16)	114	1.00 (0.79-1.25)	
19 to less than 21	1,506	1.00 (ref)	221	1.00 (ref)	
21 to less than 23	959	0.83 (0.77-0.90)	148	0.88 (0.71-1.09)	
≥23	744	0.74 (0.67-0.81)	99	0.69 (0.54-0.88)	0.16
BMI at age 18, continuous, RR (*P *trend)^f^		0.77 (<0.001)		0.78 (0.003)	0.80
Alcohol intake (g/day)					
0	2,552	1.00 (ref)	334	1.00 (ref)	
<5	876	1.15 (1.06-1.24)	131	1.30 (1.05-1.59)	
5 to less than 15	765	1.22 (1.12-1.32)	113	1.34 (1.08-1.67)	
≥15	462	1.31 (1.19-1.45)	81	1.75 (1.36-2.24)	0.20
Alcohol intake, continuous, RR (*P *trend)^g^		1.04 (<0.001)		1.07 (<0.001)	0.11
Family history of breast cancer					
No	3,805	1.00 (ref)	544	1.00 (ref)	
Yes	850	1.55 (1.44-1.67)	115	1.41 (1.15-1.73)	0.39
Personal history of benign breast disease					
No	2,294	1.00 (ref)	298	1.00 (ref)	
Yes	2,361	1.47 (1.38-1.56)	361	1.60 (1.37-1.88)	0.32

^a^Estimates adjusted for all variables presented in the table. ^b^*P *value from likelihood ratio test comparing, for each covariate, the model with separate estimates for the ductal and lobular histologic subtypes to the model with a single estimate across the two subtypes. ^c^Risk ratio (RR) for each 5-year increase in age among parous women. ^d^RR for each child among parous women. ^e^RR for each 5-year increase in use. ^f^RR for each five-unit increase in BMI (kg/m^2^). ^g^RR for each five-unit increase in daily alcohol consumption (g/day). 95% CI, 95% confidence interval.

The associations with age at menarche, AFB and PMH use differed significantly by histology (*P*-het = 0.03, <0.001 and <0.001, respectively). Age at menarche was inversely associated with risk of both ductal and lobular cancers; however, the association was stronger for lobular cancers (2% vs. 8% decrease in risk for each year increase in age at menarche, respectively).

Increasing AFB was associated with an increasing risk of both ductal and lobular cancers, although the results were stronger for lobular cancers (*P*-het < 0.001). For example, compared with the reference group (that is, parous women with an AFB <25), the RRs associated with an AFB between 30 and 34 years old were 1.21 (95% CI, 1.09-1.35) and 2.31 (95% CI, 1.79-2.99) for ductal and lobular cancers, respectively. There was significant heterogeneity when modeling AFB and parity as continuous variables (*P*-het = <0.001 and 0.02, respectively). For ductal and lobular tumors, respectively, the RRs for every 5-year increase in AFB were 1.12 (*P *trend < 0.001) and 1.48 (*P *trend < 0.001), while the RRs for each additional birth were 0.97 (*P *trend = 0.01) and 1.05 (*P *trend = 0.11).

There was significant heterogeneity for PMH use across subtypes (*P*-het < 0.001). Compared to never users, past use was not significantly associated with risk of ductal or lobular breast cancer (RR = 1.05; 95% CI, 0.97-1.14; and RR = 1.11; 95% CI, 0.88-1.39, respectively). Current use of E alone for <5 years was associated with a statistically significant increase in the risk of lobular cancer (RR = 1.56; 95% CI, 1.01-2.42) but not ductal cancer (RR = 1.07; 95% CI, 0.90-1.28), while use of E alone for 5 to 10 years was associated with an increased risk of both cancer types, albeit the risk for lobular tumors was stronger (RR = 1.30; 95% CI, 1.17-1.45; and RR = 1.76; 95% CI, 1.34-2.34, for ductal and lobular cancers, respectively). Current E+P use was associated with increased risks of both subtypes of breast cancer, although we found a stronger RR for lobular than ductal cancers within each duration category. Specifically, the RRs for <5 years of use vs. no use were 1.56 (95% CI, 1.36-1.78) for ductal cancer and 2.16 (95% CI, 1.52-3.06) for lobular cancer, while the RRs for 5-10 years of use were 1.75 (95% CI, 1.57-1.95) and 3.12 (95% CI, 2.41-4.05) for ductal and lobular cancers, respectively.

### Nonreproductive exposures

BMI was positively associated with risk of both subtypes (*P*-het = 0.20) (Table 2). Compared to women with BMI <21, women with BMI ≥30 had RRs of 1.60 (95% CI, 1.42-1.80) and 1.47 (95% CI, 1.08-2.00) for ductal and lobular cancers, respectively. In contrast, BMI at age 18 was inversely associated with risk of both subtypes (RR ≥23 vs. 19 to <21 = 0.74; 95% CI, 0.67-0.81 for ductal; and 0.69; 95% CI, 0.54-0.88 for lobular cancers; *P*-het = 0.16). For each five-unit increase in BMI at age 18, there was a significant 23% and 22% decrease in risk of ductal and lobular cancers, respectively. We [15] and others [16,17] have shown that obesity is more strongly associated with breast cancer risk among women who have never used PMH. In a supplementary analysis among never users, there was significant heterogeneity in the association between BMI and risk by tumor subtype (*P*-het = 0.02) with a stronger positive association for ductal vs. lobular tumors (RR, BMI ≥30 vs. <21 = 2.11; 95% CI, 1.71-2.60; and RR = 1.70; 95% CI, 0.97-2.98, respectively). However, the sample size was reduced (1,596 ductal and 177 lobular cases).

The positive associations for family history of breast cancer and a personal history of BBD were similar across the two subtypes (Table 2). There was suggestion of a stronger positive association between increasing daily alcohol intake and risk of lobular cancer (*P*-het = 0.11 for each five-unit increase).

### ER and PR status

Since lobular tumors are more likely to be ER-positive and PR-positive than ductal cancers, we restricted analyses to ER-positive and PR-positive tumors in secondary analyses to address whether our observations were driven by differential proportions of hormone receptor positivity between ductal and lobular tumors. A total of 2,233 tumors ER-positive and PR-positive tumors were included in this analysis (Table 3). In general, the results were similar to those we observed in the analysis among all tumors. Among the reproductive and hormonal risk factors, there were still significant differences for the associations with age at menarche, AFB and PMH use, but not for age, nulliparity, parity, age at menopause or menopausal type (*P*-het ≥ 0.16). Nevertheless, there was significant heterogeneity for the relationship with increasing parity when we modeled this exposure continuously (*P*-het = 0.05). Similar to the analysis including the entire cohort, the protective effect of parity was stronger for ductal vs. lobular tumors. For the nonreproductive exposures, there was significant heterogeneity for the association with BMI at 18 years of age (*P*-het = 0.03) and increasing daily alcohol intake (*P*-het = 0.02), with a stronger association for lobular cancers but no heterogeneity of effect for adult BMI, family history of breast cancer or history of BBD (*P*-het ≥ 0.07).

**Table 3:** Association between various reproductive and non-reproductive exposures and risk of postmenopausal breast cancer by histologic subtype among 107,759 women in the Nurses' Health Study, ER-positive and PR-positive tumors only^a^

	Ductal (*n *= 4,655)		Lobular (*n *= 659)		
Reproductive or hormonal exposures	Cases (n)	RR (95% CI)	Cases (n)	RR (95% CI)	***P*-heterogeneity**^**b**^
Age (per year)^c^	1,947	1.03 (1.02-1.04)	286	1.04 (1.02-1.06)	0.71
Age at menarche (per year)^c^	1,947	0.99 (0.96-1.02)	286	0.87 (0.80-0.95)	0.005
Parous	1,795	1.00 (ref)	276	1.00 (ref)	
Nulliparous	200	1.17 (0.94-1.47)	17	0.90 (0.47-1.72)	0.45
Age at first birth (AFB)					
AFB < 25	1,040	1.00 (ref)	111	1.00 (ref)	
AFB 25-29	730	1.06 (0.95-1.17)	131	1.82 (1.39-2.38)	
AFB 30-34	174	1.09 (0.92-1.29)	41	2.62 (1.78-3.88)	
AFB ≥35	51	1.02 (0.76-1.37)	10	2.10 (1.04-4.21)	<0.001
AFB, continuous, RR (*P *trend)^c^		1.11 (0.004)		1.56 (<0.001)	<0.001
Parity					
Parity = 1	360	1.00 (ref)	48	1.00 (ref)	
Parity = 2	476	0.82 (0.68-0.99)	72	0.71 (0.46-1.10)	
Parity = 3	545	0.90 (0.75-1.08)	69	0.71 (0.45-1.11)	
Parity ≥ 4	614	0.80 (0.67-0.97)	104	0.94 (0.61-1.46)	0.09
Parity, continuous, RR (*P *trend)^d^		0.98 (0.15)		1.06 (0.12)	0.05
Postmenopausal hormone use					
Never used	738	1.00 (ref)	90	1.00 (ref)	
Past use	549	1.09 (0.97-1.22)	73	1.18 (0.85-1.63)	
Current use, estrogen alone < 5 years	59	1.01 (0.77-1.32)	12	1.64 (0.88-3.05)	
Current use, estrogen plus progesterone < 5 years	155	1.74 (1.45-2.08)	29	2.95 (1.90-4.57)	
Current use, other ≤10 years	91	1.28 (1.03-1.59)	14	1.82 (1.05-3.17)	
Current use, estrogen alone 5-10 years	118	1.36 (1.11-1.67)	26	2.49 (1.57-3.97)	
Current use, estrogen + progesterone 5-10 years	180	2.05 (1.73-2.43)	35	3.37 (2.23-5.08)	<0.001
Estrogen alone, continuous, RR (*P *trend)^e^		1.00 (0.006)		1.00 (0.63)	0.53
Estrogen + progesterone, continuous, RR (*P *trend)^e^		1.03 (<0.001)		1.03 (<0.001)	0.24
Other, continuous, RR (*P *trend)^e^		1.00 (0.71)		1.01 (0.36)	0.44
Age at menopause (per year)^c^	1,947	1.00 (1.00-1.01)	286	1.02 (1.00-1.03)	0.16
Menopausal type					
Natural or radiation-induced	1,337	1.00 (ref)	185	1.00 (ref)	
Surgical with two ovaries removed	194	0.75 (0.64-0.88)	29	0.89 (0.59-1.36)	
Surgical with one or no ovaries removed	202	0.86 (0.73-1.00)	35	1.10 (0.75-1.60)	0.63
Nonreproductive exposures					
Body mass index (BMI)					
<21	178	1.00 (ref)	26	1.00 (ref)	
21 to less than 23	251	1.05 (0.87-1.27)	37	1.08 (0.66-1.78)	
23 to less than 25	347	1.27 (1.06-1.52)	51	1.32 (0.82-2.11)	
25 to less than 30	677	1.54 (1.30-1.82)	98	1.49 (0.96-2.31)	
≥30	465	2.00 (1.67-2.39)	67	1.87 (1.16-3.01)	0.23
BMI, continuous, RR (*P *trend)^f^		1.23 (<0.001)		1.25 (<0.001)	0.79
BMI at age 18					
<19	355	1.18 (1.04-1.35)	51	1.42 (0.97-2.01)	
19 to less than 21	593	1.00 (ref)	76	1.00 (ref)	
21 to less than 23	399	0.84 (0.74-0.95)	71	1.22 (0.89-1.67)	
≥23	354	0.81 (0.71-0.93)	49	0.89 (0.61-1.30)	0.03
BMI at age 18, continuous, RR (*P *trend)^f^		0.80 (<0.001)		0.81 (0.08)	0.95
Alcohol intake (g/day)					
0	1,062	1.00 (ref)	148	1.00 (ref)	
<5	358	1.16 (1.03-1.31)	47	1.11 (0.80-1.55)	
5 to less than 15	341	1.40 (1.23-1.58)	51	1.52 (1.10-2.10)	
≥15	186	1.37 (1.17-1.61)	40	2.28 (1.60-3.24)	0.07
Alcohol intake, continuous, RR (*P *trend)^g^		1.05 (<0.001)		1.12 (<0.001)	0.02
Family history of breast cancer					
No	1,553	1.00 (ref)	227	1.00 (ref)	
Yes	394	1.71 (1.53-1.91)	59	1.80 (1.35-2.39)	
Personal history of benign breast disease					0.76
No	972	1.00 (ref)	142	1.00 (ref)	
Yes	975	1.43 (1.30-1.56)	144	1.41 (1.10-1.78)	0.91

^a^Estimates adjusted for all variables presented in the table. ^b^*P *value from likelihood ratio test comparing, for each covariate, the model with separate estimates for the ductal and lobular histologic subtypes to the model with a single estimate across the two subtypes. ^c^Risk ratio (RR) for each 5-year increase in age among parous women. ^d^RR for each child among parous women. ^e^RR for each 5-year increase in use. ^f^RR for each five-unit increase in BMI (kg/m^2^). ^g^RR for each five-unit increase in daily alcohol consumption (g/day).

## Discussion

The results from this large prospective study further substantiate that well-established breast cancer risk factors differentially affect risk on the basis of tumor histology. Specifically, age at menarche, AFB and PMH use were more strongly associated with lobular versus ductal tumors, while there was no evidence for heterogeneity for age at menopause, nulliparity, parity, menopausal type, alcohol consumption, adult BMI, BMI at age 18, family history of breast cancer and a personal history of BBD. Collectively, these data suggest different etiologies among the tumor subtypes and that lobular carcinomas may represent a more hormonally responsive subtype. When our analysis was limited to ER-positive and PR-positive tumors, the findings were maintained, suggesting true differences in the underlying tumor biology and not a result of differential rates of ER and PR positivity.

A recent prospective analysis examined reproductive and hormonal factors and risk of breast cancer by histology among 17,923 ductal and 3,332 lobular cancers from the Million Women Study [18]. Similar to our results, the effect of age at menarche was greater for lobular compared with ductal tumors (RR per 5-year increase = 0.65 for lobular and 0.93 for ductal). Further, in the Reeves *et al*. [18] meta-analysis of six cohort and three case control studies, the summary estimates were similar. No formal tests for heterogeneity were performed. Although suggestive of differences in the effect of age at menarche by histologic subtype, most studies had low power and only one study was restricted to postmenopausal women [19]. Overall, prior studies and our observations support a stronger protective effect of a later age at menarche for lobular cancer.

The overall RRs for AFB ≥30 vs. <20 from a meta-analysis of nine studies (six cohort and three case control) were 1.24 for ductal cancer and 1.66 for lobular cancer; however, individually, most studies had limited power, and only the Million Women Study reported a statistically significant difference in the RRs by subtype [18]. Phipps *et al*. [20] recently reported no difference in risk with AFB across subtypes. In our analysis, nulliparity was associated with an increased risk of both cancer subtypes, while we observed significant heterogeneity in the association only for AFB, but not for parity *per se*. When we modeled the continuous values, the *P *trend for increasing parity was significant only for ductal tumors; however, the effect of AFB was strongest for lobular tumors. We cannot preclude that the discrepancy between the categorical and continuous results for parity may be attributed to residual confounding by AFB, which does not occur when modeled as categorical or nonoverlapping variables. In other words, it may be that women with higher parity are also likely to have a later AFB. Our RR estimates for a 5-year delay in AFB were 1.48 for lobular cancer vs. 1.12 for ductal cancer. Other studies have reported no difference in risk for parity by subtype [18,21,22]. Prior reports are in agreement with our findings regarding parity. A recent prospective analysis including 1,211,238 women reported a lower risk of ductal but not lobular cancers with parity [20]. Two additional reports have suggested that increasing parity was associated with decreased risk of ductal but not lobular cancers, although one study did not report *P *values [23] and the association was not statistically significant in the second study [24]. On the basis of our data and prior data, a later AFB and not parity *per se *may play an important etiological role for lobular cancers, while both AFB and number of births appear to be important for ductal carcinomas. Similar to our findings, most prior reports of the relationship between age at menopause or menopausal status and risk have reported no significant heterogeneity by histology [18,21,23,25,26].

To date, 10 case control and 7 cohort studies have examined the relationship between PMH use and risk of developing ductal and lobular carcinoma [7,20]. Of these, all but two cohort studies reported a stronger increased risk for lobular compared with ductal carcinoma [27,28]. In general, most studies have shown that combined E+P was more strongly related to risk of lobular vs. ductal carcinoma than use of E alone [7,29]. Lobular cancers are more likely to be ER-positive and/or PR-positive than ductal tumors [30,31]. Thus, it is not surprising that the effect of PMH may be strongest for a hormone receptor-positive tumor. In fact, a recent study reporting a decrease in the incidence of invasive cancers, particularly a decline in lobular carcinomas [32], attributed this change to the concurrent decrease in PMH use [33] following publication of the results from the Women's Health Initiative [34]. Unlike what we observed among all tumors, the association with PMH did not differ significantly by histologic subtype when restricted to ER-positive and PR-positive lobular and ductal tumors; however, this could be attributed to the smaller case numbers, since the association was still suggestively stronger for lobular tumors.

Fewer studies have examined the relationship between duration of hormone use and risk [7,23,29,35-38]. In the Million Women Study, Reeves *et al*. [29] reported no significant difference in risk of ductal, lobular or tubular cancer with increasing duration of use. Results from the French E3N cohort also suggested no significant heterogeneity [38]. Calle *et al*. [7] reported an increased risk with ≥2 years of use among current users for both ductal and lobular cancers. In contrast, E alone was associated with an increased risk of lobular cancer after 5 years of use, but not with risk of ductal cancer; however, use of E alone for 5-10 years was associated with risk of both cancer types, although the association was stronger for lobular tumors. Similarly, Li *et al*. [35] documented an increased risk of both histologic subtypes with ≥3 years of use of E+P. Other case control studies have also found an increased risk of lobular cancer with longer duration of combined E+P use [10,36,37]. With our large study population and repeated assessment of PMH use, our study is adequately powered to assess a role of both timing and duration of PHH use. We similarly observed a stronger association with use of combined E+P and the lobular type. Although there was no significant heterogeneity in the results when we modeled PMH duration as a continuous variable, this was expected, since we previously reported that breast cancer risk is increased only with long-term use (for example, at least 5 years) [39]. Nonetheless, others have shown a significant increase in risk with shorter durations of use [7,28,40].

Epidemiological studies have demonstrated a positive association between BMI and postmenopausal breast cancer risk [41], although for the most part anthropometric factors appear to be similarly related to risk by histology in some [8,25] but not all studies [42]. Despite this, BMI was more strongly associated, although not statistically different, with ductal cancer compared with lobular cancer in one study [21], while another group reported an inverse association between BMI and mixed ductal-lobular carcinomas (*P*-het = 0.008), but no significant associations for the other subtypes [26]. We did not observe significant heterogeneity for the association between BMI and risk of cancer by subtype.

To our knowledge, ours is the first study that has evaluated an association between BMI at age 18 and risk of postmenopausal breast cancer by histologic subtype. In general, adolescent obesity is inversely associated with breast cancer risk [41]. In a prospective analysis of the NHS, we previously reported that higher BMI at age 18 was associated with lower breast cancer incidence prior to and after menopause [15]. In the current study, the inverse association between BMI at 18 was similar for ductal and lobular cancers.

Two prior studies reported a significant positive association between alcohol consumption and risk of breast cancer overall, which was stronger for lobular compared with ductal cancer among postmenopausal women (odds ratio (OR) = 1.8 vs. 1.2 [43] and 1.5 vs. 1.2 [26] for lobular and ductal cancers, respectively). In a third study, Rosenberg *et al*. [23] found a strong association between alcohol consumption and risk of tubular but not ductal or lobular cancers. Although we did not observe significant heterogeneity between daily alcohol consumption and risk overall, we did observe a positive association between increasing intake and risk which was suggestively stronger for lobular cancers (*P*-het = 0.11). Despite these findings, there was significant heterogeneity for this association when we limited the analysis to ER-positive and PR-positive tumors (*P*-het = 0.02). Given the inconsistent results, further research is needed for this exposure.

In our analysis, family history of breast cancer and a personal history of BBD were similarly associated with increased risk of breast cancer, regardless of subtype. Three prior studies have evaluated the relationship between family history and risk by histology [9,20,26]. Recently, Phipps *et al*. [20] reported similar positive associations across subtypes in their cohort study. In a population-based case control analysis, family history was associated with a twofold increased risk of breast cancer across all histologic subtypes except for mucinous tumors [26], while family history was similarly associated with a two- to threefold increase in risk of all subtypes except for tubular carcinomas [9]. No confidence intervals or formal tests for heterogeneity were reported in the latter study. To our knowledge, two prior studies have evaluated the relationship between BBD and breast cancer risk stratified by histologic subtype [20,23]. A previous operation for BBD was associated with a slightly higher risk of lobular cancer (OR = 1.9) compared with ductal cancer (OR = 1.5), although this difference did not achieve statistical significance. In contrast, a recent cohort study found similar significant positive associations with BBD across subtypes with increasing density (hazard ratio for highest density vs. reference for both ductal and lobular cases was 1.62) [20]. On the whole, there do not appear to be large differences in the relationship between family history of breast cancer or personal history of BBD by histology.

Overall, the relationship between the exposures evaluated in the current study and the risk of breast cancer was within the expected range and both subtypes showed similar directions; however, the associations for age at menarche, AFB and PMH use were stronger for risk of lobular vs. ductal tumors. Since lobular tumors are more often hormone receptor-positive, it is not entirely unexpected that various hormonal or reproductive risk factors may be more strongly associated with this subtype. The proposed mechanism underlying the association between an earlier age at menarche and risk likely is via its effect on the number of lifetime ovulatory cycles, which in turn influences lifetime exposure to endogenous ovarian hormones [44]. Parity not only leads to substantial changes in hormone levels but also induces differentiation of terminal duct lobular units, making the breast epithelial cells less susceptible to carcinogenic insult [45]. Given that both exogenous and endogenous hormones have been shown to strongly influence breast cancer risk, an altered sex hormone profile associated with PMH use as an exogenous hormone source would also be expected to increase risk [2]. Collectively, the data suggest that lobular breast tumors are more hormonally responsive than ductal breast tumors, thus making this histologic subtype more responsive to hormonal stimulation [9]. Furthermore, exposure to reproductive hormones (that is, estrogen or progesterone) has been shown to promote lobular rather than ductal differentiation, subsequently increasing the number of lobular cells at risk [9]. In addition, various groups have shown differences in gene expression between these two histologic subtypes, providing insight into the biology of these tumors, which may in turn be utilized as diagnostic or therapeutic targets [46,47]. Future studies should continue to investigate the biological basis for the reported differential relationships.

Major strengths of our study include the large number of participants and high participation rate, resulting in adequate statistical power to conduct stratified analyses. Further, the prospective nature of the NHS cohort allows for the analysis of repeated measures of most exposures and confounders, limiting the effect of measurement error. In addition, the methods used in this analysis allowed for the estimation of separate associations with each subtype simultaneously, as well as formal tests for differences in risk across subtypes. Although our analysis included a large number of invasive breast cancer cases, we did not have enough cases to assess risk with less common histologic subtypes (for example, tubular). Further, our analysis of ER-positive and PR-positive tumors was less well-powered, given that hormone receptor status was not available for all cases. An additional limitation was that we classified histologies on the basis of a review of pathology reports and did not conduct a centralized pathology review. This could lead to nondifferential misclassification of tumor histology and attenuate the associations.

## Conclusions

In summary, the results from this study provide evidence that some reproductive or hormonal and nonreproductive factors are differentially associated with risk of postmenopausal breast cancer on the basis of histologic subtype. For the most part, our results are consistent with prior reports, complementing the growing body of evidence suggesting etiologic heterogeneity between ductal and lobular tumors. Future risk factor analyses should continue to investigate associations stratified by histology.

## Abbreviations

AFB: age at first birth; BBD: benign breast disease; BMI: body mass index; CI: confidence interval; DCIS: ductal carcinoma *in situ*; E: estrogen; E + P: estrogen and progesterone; ER: estrogen receptor; NHS: Nurses' Health Study; OR: odds ratio; *P*-het: *P *value for heterogeneity; PMH: postmenopausal hormones; PR: progesterone receptor; RR: relative risk.

## Competing interests

The authors declare that they have no competing interests.

## Authors' contributions

JK helped to design, analyze and interpret the data and drafted the initial manuscript. WYC helped to design, analyze and interpret the data and did critical revisions of the manuscript. MAG helped with statistical analysis and data interpretation. SEH and SST both helped analyze and interpret the data and critically revised the manuscript. BAR helped with statistical analysis, interpreted the data and critically revised the manuscript. All authors read and approved the final manuscript.
